# The Pyroptosis-Related Long Noncoding RNA Signature Predicts Prognosis and Indicates Immunotherapeutic Efficiency in Hepatocellular Carcinoma

**DOI:** 10.3389/fcell.2022.779269

**Published:** 2022-05-26

**Authors:** Tao Wang, Yi Yang, Ting Sun, Haizhou Qiu, Jian Wang, Cheng Ding, Ren Lan, Qiang He, Wentao Wang

**Affiliations:** ^1^ Department of Liver Surgery and Liver Transplantation Center, West China Hospital of Sichuan University, Chengdu, China; ^2^ State Key Laboratory of Biotherapy and Cancer Center, West China Hospital, Sichuan University, Chengdu, China; ^3^ Department of Hepatobiliary Surgery, Beijing Chaoyang Hospital Affiliated to Capital Medical University, Beijing, China

**Keywords:** pyroptosis, long non-coding RNAs, hepatocellular carcinoma, prognosis, immunity

## Abstract

Pyroptosis was recently demonstrated to be an inflammatory form of gasdermin-regulated programmed cell death characterized by cellular lysis and the release of several proinflammatory factors and participates in tumorigenesis. However, the effects of pyroptosis-related long noncoding RNAs (lncRNAs) on hepatocellular carcinoma (HCC) have not yet been completely elucidated. Based on the regression coefficients of ZFPM2-AS1, KDM4A-AS1, LUCAT1, NRAV, CRYZL2P-SEC16B, AL031985.3, SNHG4, AL049840.5, AC008549.1, MKLN1-AS, AC099850.3, and LINC01224, HCC patients were classified into a low- or high-risk group. The high-risk score according to pyroptosis-related lncRNA signature was significantly associated with poor overall survival even after adjusting for age and clinical stage. Receiver operating characteristic curves and principal component analysis further supported the accuracy of the model. Our study revealed that a higher pyroptosis-related lncRNA risk score was significantly associated with tumor staging, pathological grade, and tumor-node-metastasis stages. The nomogram incorporating the pyroptosis-related lncRNA risk score and clinicopathological factors demonstrated good accuracy. Furthermore, we observed distinct tumor microenvironment cell infiltration characteristics between high- and low-risk tumors. Notably, based on the risk model, we found that the risk score is closely related to the expression of immune checkpoint genes, immune subtypes of tumors, and the sensitivity of HCC to chemotherapy drugs and immunotherapy. In conclusion, our novel risk score of pyroptosis-related lncRNA can serve as a promising prognostic biomarker for HCC patients and provide help for HCC patients to guide precision drug treatment and immunotherapy.

## Introduction

Liver cancer is a highly heterogeneous malignancy and is considered the fourth most common cause of cancer-related death worldwide with considerable number of new cases and deaths due to hepatocellular carcinoma (HCC) each year (Bray et al., 2018; Fitzmaurice et al., 2019; Yang et al., 2019). HCC is the most common histological type of liver cancer and is associated with poor prognosis. The incidence of HCC is rising worldwide because of the increasing prevalence of viral hepatitis, alcoholism, and nonalcoholic steatohepatitis (Forner et al., 2018; Yang et al., 2019). In the past few years, despite great progress in cancer molecular biology and precision techniques including minimally invasive surgery, radiation, chemotherapy, TACE, and molecular targeted drugs, and so on, the long-term prognosis of HCC patients remains poor because of the high recurrence rate and intrahepatic or extrahepatic metastasis (Zhu et al., 2020). Considering the high mortality rate of HCC patients, new molecular biomarkers with diagnostic and prognostic significance and development of novel therapeutic targets could improve the clinical prognosis.

Pyroptosis, also known as cytoinflammatory necrosis, is a newly discovered form of programmed cell death related to inflammation, and it has morphological characteristics of both apoptosis and necrosis (Shi et al., 2017; Zhou et al., 2020a). Pyroptosis can cause cell swelling, plasma membrane lysis, chromatin fragmentation, and the release of intracellular proinflammatory content, which is characterized by the production of NLRP3 inflammasomes and the release of inflammatory cytokines (interleukin-1β, interleukin-18). Current research found that the induction pathways of pyroptosis mainly include the classical pyroptosis pathway that depends on caspase-1 and the nonclassical pyroptosis pathway that depends on caspase-4/5/11, the apoptosis-pyroptosis transformation pathway caused by high expression of gasdermin E (GSDME) (Kayagaki et al., 2011; Miao et al., 2011). Past studies have shown that pyroptosis is essential for the body to resist pathogen infection. In recent years, its anticancer effect has gradually attracted wide attention from scholars at home and abroad. Studies have found that when pyroptosis occurs, the body will produce the strong inflammatory response, and the body’s immune system will also be activated, it may also affect the tumor immune microenvironment and then effectively eliminate malignant cells and boost anticancer immunity (Tang et al., 2020; Wang et al., 2021a).

In addition, various inflammatory cytokines released in the process of pyroptosis might be closely related to the occurrence of tumors, the sensitivity of immunotherapy, and the resistance to chemotherapeutics (Fang et al., 2020; Ren et al., 2020). However, there is a lack of a comprehensive understanding of the mechanism and function of pyroptosis-related genes in HCC on tumor progression and metastasis.

Long noncoding RNAs (lncRNAs) are RNAs with a length of more than 200 nucleotides that are not involved in protein translation (Statello et al., 2021). However, they can affect cell proliferation, cell cycle, differentiation, and apoptosis by participating in gene regulation (Goodrich and Kugel, 2006; Rinn et al., 2007; Yoon et al., 2013). Increasing transcriptome sequencing of tumors has revealed thousands of lncRNAs, whose abnormal expression is associated with various types of cancers and plays an important role in tumorigenesis and tumor prognosis. Therefore, identifying ideal lncRNAs as biomarkers is expected to become a new approach for precision treatment.

Based on the findings, pyroptosis, as a mechanism of cell death, can inhibit tumor cell proliferation, invasion, and metastasis, thereby playing an important role in the cancer development (Fang et al., 2020). In addition, differential expression of lncRNAs and abnormal alterations in pyroptosis are both common phenomena in tumor cells. However, their direct interconnection and role in HCC are unknown and worthy of further investigation. Thus, we performed this comprehensive systematic study to determine the differential expression of pyroptosis-related lncRNA using The Cancer Genome Atlas (TCGA) databases and developed a pyroptosis-related lncRNA signature with prognostic value to provide promising biomarkers. Meanwhile, we evaluated the immune function and immune status between the high- and low-risk groups associated with pyroptosis-related lncRNA signatures, as well as the response to chemotherapy and immunotherapy. The results of this study will provide an in-depth understanding of the regulatory mechanism of pyroptosis in HCC and provide help for individualized interventions and treatments for patients with HCC.

## Materials and Methods

### Dataset Extraction and Pyroptosis-Related Gene Detection

**FIGURE 1:**
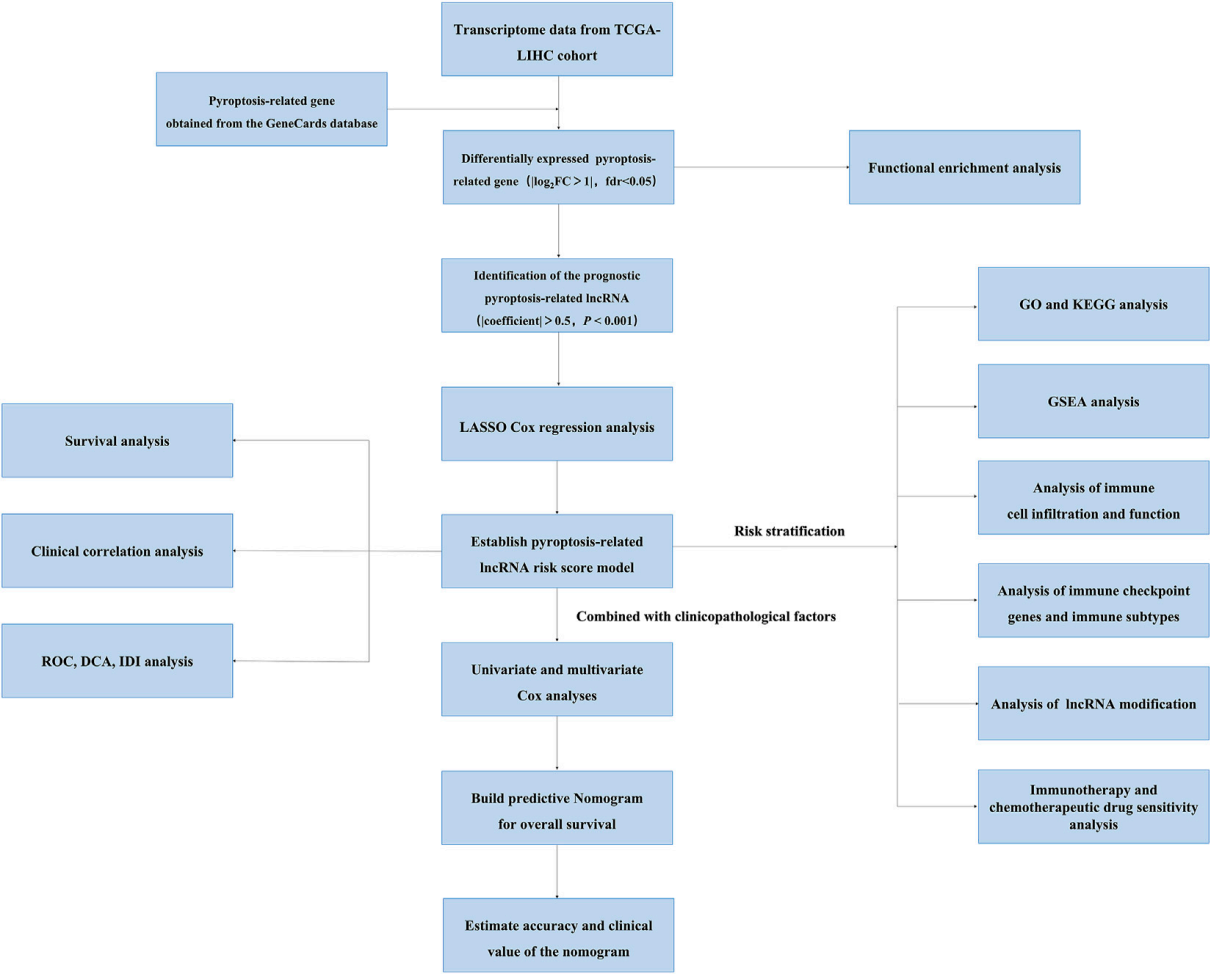
The flowchart of this study showing the process of constructing the 12 pyroptosis-related lncRNA risk score to predict prognosis of HCC.

We obtained the RNA sequencing (RNA-seq) expression data of 370 HCC patients and the corresponding clinical data from TCGA database on August 1, 2021 (https://portal.gdc.cancer.gov/repository). The flowchart of the present study design is shown in Figure 1. Pyroptosis-related genes were obtained from the GeneCards database (August 1, 2021. https://www.genecards.org/) using the keywords “pyroptosis” and from reviewing and summarizing previous literature (Shi et al., 2017; Karki and Kanneganti, 2019; Xia et al., 2019; Zhang et al., 2020a; Fang et al., 2020; Huang et al., 2021a; Fu et al., 2021). In total, 206 pyroptosis-related genes are listed in Supplementary Table S1. lncRNA and mRNA expression profiles were extracted from the TCGA database, and then, pyroptosis-related lncRNAs were obtained from pyroptosis-related genes by calculating the coexpression correlation coefficient (|coefficient|> 0.50, *p* < 0.001). Pyroptosis genes and pyroptosis-related lncRNA regulatory networks were built using Cytoscape software (version 3.8.0). The “limma” package of R software (version 4.1.0) was used to identify differentially expressed pyroptosis-related genes and pyroptosis-related lncRNAs. Criteria were established by |log_2_ fold change|≥ 1, while adjusted *p* < 0.05 indicated significant statistical differences.

### Gene Set Enrichment Analysis

After determining the significant differentially expressed pyroptosis-related lncRNAs between the subgroups categorized by the risk model, the Gene Ontology (GO) and Kyoto Encyclopedia of Genes and Genomes (KEGG) pathways were used to evaluate the biological function of the prognostic candidates by the “clusterProfiler” R package by applying the criteria false discovery rate <0.05 and |log_2_ fold change (FC)| ≥ 1. Gene set enrichment analysis (GSEA) was used to compare the biological processes that were significantly changed between the high- and low-risk groups by GSEA software, and the gene set “c2. cp.kegg.v7.4. symbols.gmt” from the KEGG database was chosen as the reference.

### Identification of a Prognostic Pyroptosis-Related LncRNA Model for HCC

The “limma” package of R software was used to identify differentially expressed pyroptosis-related genes and the pyroptosis-related lncRNAs. Criteria were established by |log_2_FC| ≥1, whereas adjusted *p* < 0.05 indicated significant statistical differences. Univariate Cox regression was applied to identify pyroptosis*-*related lncRNAs related to overall survival (OS) for model construction (*p *< 0.05). Then, to narrow down the candidate pyroptosis-related lncRNA and synthetically estimate the significant values, the LASSO Cox regression algorithm (R package “glmnet”) was used to construct a formula for each patient: formula = expressionindex1× *β*index1+…+expressionindexn × *β*indexn (where *β* is the regression coefficient derived from LASSO regression). HCC patients were divided into high- and low-risk groups by the median risk score. The difference in lncRNAs between the high- and low-risk groups and the association patterns distributed between the clinical–pathological characteristics and the risk score are displayed in the form of a heatmap using the R package of “pheatmap.” Survival analysis was performed between the high- and low-risk groups using Kaplan–Meier methods. The area under the time-dependent receiver operating characteristic (ROC) curve associated with 1-, 2-, and 3-year survival was applied to evaluate the predictive ability of the above-identified lncRNAs. The risk curve was used to explore differences in survival status between different risk groups of patients.

### Independent Prognostic Analysis

To evaluate whether the pyroptosis-related risk score is an independent factor affecting the survival of HCC patients, univariate analysis was performed on the TCGA dataset. We constructed the nomogram integrating gender, grade, age, stage, and pyroptosis-related risk score using the “regplot” package. The calibration curve was used to evaluate the predictive accuracy for the nomogram of 1-, 3-, and 5-year OS. Integrated discrimination improvement (IDI) was used to quantify the improvement in sensitivity and specificity of our pyroptosis-related lncRNA signature compared with the previously reported lncRNA prediction model for HCC. The clinical benefit was measured by using the decision curve analysis (DCA).

### Immunogenomic Landscape Analyses

The currently acknowledged computational methods, including TIMER (Li et al., 2020), CIBERSORT (Chen et al., 2018), CIBERSORT-AB (Luan et al., 2021), QUANTISEQ (Plattner et al., 2020), MCPCOUNT (Zhang et al., 2020b), XCELL (Aran et al., 2017), and EPIC (Racle et al., 2017), were used to calculate the immune infiltration score between different risk score groups. Single-sample GSEA (ssGSEA) with the “gsva” package was implemented to calculate the score of 16 infiltrating immune cells and the 13 activities of immune-related pathways (Rooney et al., 2015). The immune infiltration and functions were compared between the high- and low-risk groups using the two-sample Wilcoxon test. Immune checkpoint expression has become a biomarker for various malignancies to choose immunotherapy and improve survival prediction. Therefore, further correlation analysis was conducted between the pyroptosis-related risk score and common key immune checkpoint genes, including PD-L1 (CD274), CTLA4, TIGIT, LAG3, IDO1, TDO2, CD276, VSIR, HAVCR2, and PDCD1. We used the circular plot to show the correlation between risk score and immune checkpoint expression with the “circlize” package. Furthermore, the boxplot was used to display the differences in immune checkpoint expression between the high- and low-risk groups. In addition, we also obtained the profile of immune subtypes for HCC patients in the TGGA database from UCSC-Xena (https://xenabrowser.net/datapages/) (Thorsson et al., 2018).We compared the immune subtypes between different risk groups using the package “RColorBrewer.”

### Correlations Between the Risk Score Model and the Expression of RNA Modifications Genes

In recent years, it has been proposed that RNA modifications, including N6-methyladenosine (m^6^A) modification, 5-methylcytosine (m^5^C), and N1-methyladenosine (m^1^A), fine tune the chemostructural features of infrastructural RNAs. Increasing evidence has shown that RNA modifications play an important role in inflammation, innate immunity, and antitumor activity (Chen et al., 2021).Therefore, we compared the expression of key RNA modification genes retrieved from previous literature (Safra et al., 2017; Yang et al., 2017; Dai et al., 2018; Bohnsack et al., 2019; Zaccara et al., 2019), among different risk groups.

### Correlations Between Risk Score and Drug Treatment and Immunotherapy

To further evaluate the relationship between our pyroptosis-related lncRNA prognostic model and drug susceptibility in HCC treatment, we conducted drug susceptibility analyses with the “pRRophetic” and “ggplot2” packages to compare the half-maximal inhibitory concentration (IC_50_) of various conventional antitumor drugs for HCC between the high- and low-risk groups using the Wilcoxon signed-rank test (Liu et al., 2021a; Liu et al., 2021b; Liu et al., 2021c). The Tumor Immune Dysfunction and Exclusion (TIDE) algorithm can predict the immune checkpoint blockade response by simulating two main mechanisms of tumor immune evasion (Jiang et al., 2018). Therefore, the TIDE algorithm was used to predict potential immune checkpoint blockade responses in HCC.

## Results

### Identification of a Prognostic Pyroptosis-Related Long Noncoding Signature

**FIGURE 2:**
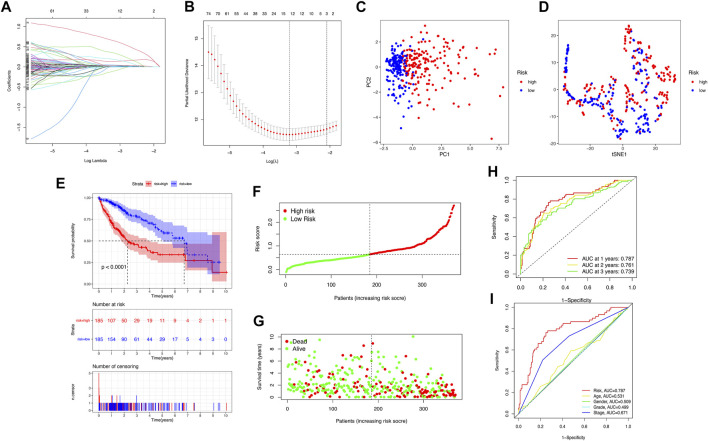
Construction of the pyroptosis-related lncRNA prognostic model for predicting prognosis of HCC patients. **(A)** Twelve pyroptosis-related lncRNA signatures were selected by LASSO Cox models. **(B)** Cross-validation for tuning parameter selection in the LASSO model. **(C,D)** Principal component analysis (PCA) and t-distributed stochastic neighbor embedding (t-SNE) analysis between the high- and low-risk groups. **(E)** The Kaplan–Meier curve survival analysis between the high- and low-risk groups. **(F,G)** The risk score distribution and survival status distribution of HCC patients in two risk groups. **(H)** ROC analysis for OS prediction including 1, 2, and 3 years of HCC patients in the training set. **(I)** ROC curve analysis compares the predictive power of risk score and other clinicopathological parameters.

We downloaded 424 sample data from the TCGA database, including 374 HCC tumor samples and 50 normal samples. First, 206 pyroptosis-related genes (mRNAs) were screened, and their expression data were available. We compared the expression levels of 206 pyroptosis-related genes between tumor tissues and normal samples, and 58 pyroptosis-related differentially expressed genes (DEGs) were identified (all *p *< 0.05), as shown in Supplementary Figure S1A. Then, the potential interactions among these pyroptosis-related genes were analyzed by the PPI network, and the results revealed that CASP3, CASP8, PYCARD, IL1B, SQSTM1, and BIRC3 were identified as core genes in the pyroptosis process of HCC (the minimum required interaction score for the PPI analysis was set at 0.9; Supplementary Figure S1B). The correlation network containing all pyroptosis-related DEGs is presented in Supplementary Figure S1C. Then, 350 pyroptosis-related lncRNAs were identified through correlation analysis (∣cor | >0.5, *p* < 0.001). Among the 89 pyroptosis-related lncRNAs related to the prognosis of HCC patients identified by univariate Cox regression analysis (*p* < 0.05), there were two pyroptosis-related lncRNA suggested to be favorable protective factors, and the remaining 87 were risk factors for the survival of HCC patients (Supplementary Figure S2). Subsequently, the risk score was calculated for each patient based on personalized lncRNA levels using LASSO Cox regression analysis based on the obtained 12 pyroptosis-related lncRNA expression profiles, shown in Figure 2A. The coefficient profiles of the selected LncRNA related to prognosis is shown in Supplementary Table S2. The pyroptosis-related lncRNAs risk score was based on the personalized levels of the 12 genes, where risk score = (0.0226 × ZFPM2-AS1) + (0.3518 × KDM4A-AS1) + (0.0472 × LUCAT1) + (0.0070 × NRAV) − (0.1110 × CRYZL2P-SEC16B) + (0.1310 × AL031985.3) + (0.0652 × SNHG4) + (0.0229 × AL049840.5) − (0.0063 × AC008549.1) + (0.6564 × MKLN1-AS) + (0.0330 × AC099850.3) + (0.1888 × LINC01224). The patients were split into low-risk (n = 185) and high-risk groups (n = 185) according to the median risk point. Principal component analysis and t-distributed stochastic neighbor embedding analysis indicated that the pyroptosis-related lncRNA risk model has good discrimination (Figures 2C,D). Kaplan–Meier analysis indicated that patients in the high-risk group had significantly poorer OS than those in the low-risk group (log-rank *p* < 0.001, Figure 2E). The estimated 1-, 3-, and 5-year OS rates were 57.84%, 15.68%, and 5.95% for the high-risk group and 83.24%, 32.97%, and 15.68% for the low-risk group, respectively (Figure 2E). Risk curve analysis showed that, compared with the low-risk group, the high-risk group had higher mortality and shorter survival times. The number of patients in the high-risk group grew, and mortality events increased with increasing risk score (Figures 2F,G). In addition, the predictive accuracy of the risk score was assessed by the time-dependent ROC analysis at 1, 2, and 3 years, with area under the curve (AUC) values of 0.787, 0.761, and 0.739, respectively (Figure 2H), Furthermore, the AUC value of OS for the risk score was significantly higher than those for age, sex, tumor stage, tumor pathological grade, and tumor staging (Figure 2I, Supplementary Figure S3). DCA curve and IDI were used to compare the discriminative ability between our model and the previously reported lncRNA prediction model for HCC (Li et al., 2020; Guo et al., 2021a; Guo et al., 2021b; Huang et al., 2021b; Wang et al., 2021b; Lei et al., 2021; Li et al., 2021). DCA indicated that our pyroptosis-related lncRNA signature had positive net benefits and superiority compared with the previously reported lncRNA prediction signature for HCC (Supplementary Figure S4). The results from IDI showed that our pyroptosis-related lncRNA signature improves the prediction performance of the status of OS for HCC patients (Supplementary Table S3).

### Correlations Between the Risk Score and Clinicopathological Factors

**FIGURE 3:**
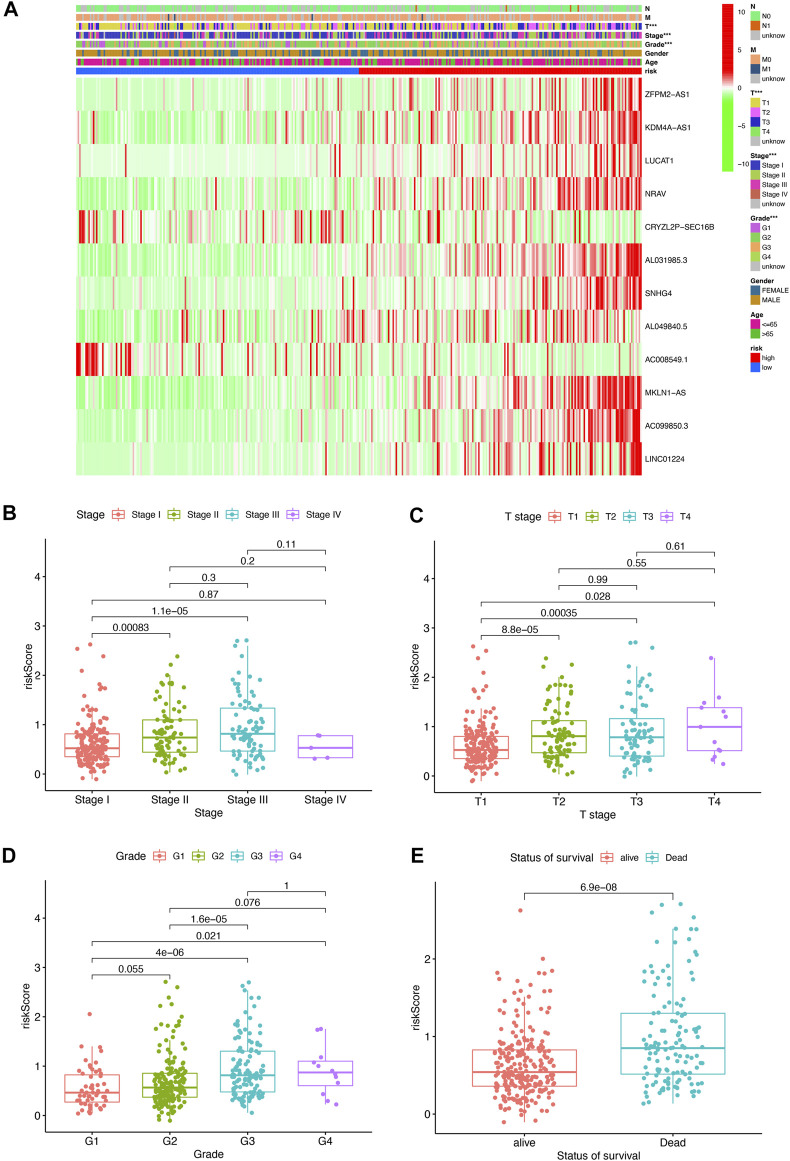
Pyroptosis-related lncRNA risk score was associated with the clinicopathological characters of patients with HCC. **(A)** Heatmap for pyroptosis-related lncRNA risk score and clinicopathological manifestation. **(B)** Boxplot of pyroptosis-related lncRNA risk score in patients with different stage. **(C)** Boxplot of pyroptosis-related lncRNA risk score in patients with different tumor stage. **(D)** Boxplot of pyroptosis-related lncRNA risk score in patients with different pathological grade. **(E)** Boxplot of pyroptosis-related lncRNA risk score in patients with different status of survival.

To further assess the roles of pyroptosis-related lncRNAs in the development of HCC, a heatmap was generated to illustrate the correlations between the risk score and clinicopathological factors including grade, stage, gender, M status, N status, and T status. We found that there were significant correlations between the risk score and pathologic grade stage, T status, and tumor stage (Figure 3A, *p* < 0.05), with a higher stage level correlating with a higher risk score. The risk score in stage I was significantly lower than that in stages II and III (Figure 3B); meanwhile, the risk score in T1 stage was significantly lower than T2, T3, and T (Figure 3C), and the above risk score was positively correlated with tumor pathological grade and survival status (Figures 3D,E). These results indicate that the pyroptosis-related lncRNA risk score is closely associated with HCC progression and prognosis.

### Assessment of Independent Prognostic Value of Risk Score

**FIGURE 4:**
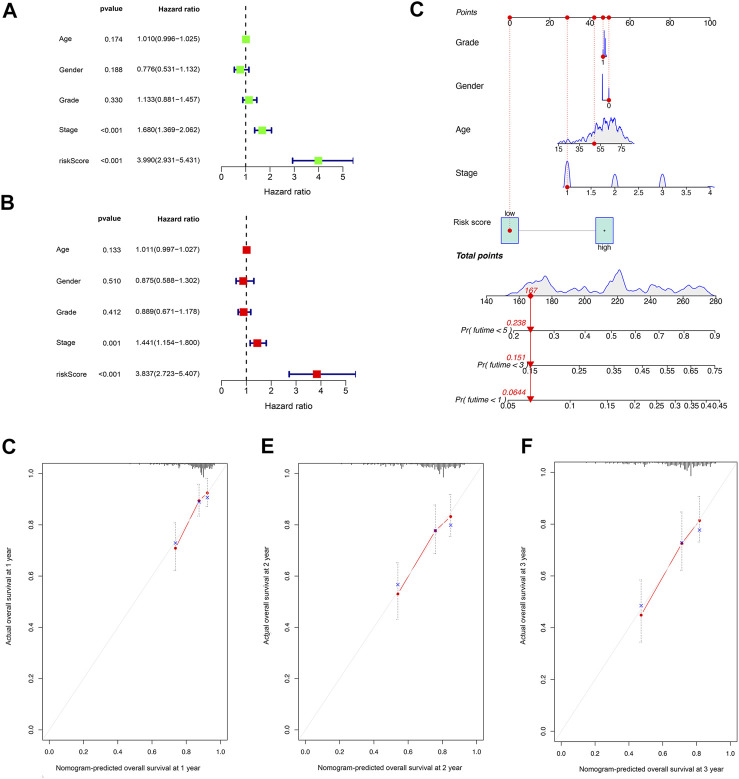
Establishment and assessment of the nomogram for survival prediction. **(A,B)** Univariate and multivariate Cox regression analyses showed that the lncRNA risk score associated with pyroptosis is an independent prognostic factor affecting the prognosis of HCC patients. **(C)** The nomogram combined pyroptosis-related lncRNA risk score and other clinicopathological parameters was developed to predict 1-, 3-, and 5-year survival. Calibration curves showing the nomogram that we established predictions for 1-year **(D)**, 2-year **(E)**, and 3-year **(F)** survival.

Univariate Cox regression analysis indicated that the risk score was an independent predictor of survival in HCC patients (hazard ratio [HR] = 3.990, 95% confidence interval [CI] = 2.931–5.431, *p *< 0.001). The multivariate Cox regression analysis identified that, even after adjusting for other confounding factors, the risk score was a prognostic factor (HR = 3.837, 95% CI: 2.723–5.407, *p *< 0.001) for HCC patients (Figures 4A,B). Subsequently, according to the stepwise Cox regression model, we established a clinically adaptable nomogram using pyroptosis-related lncRNA risk score combined with clinicopathological factors. Our nomogram displayed better accuracy in predicting both short- and long-term survival (Figure 4C). The predictive performance of this nomogram was further evaluated using the ROC curve, and the results showed a higher C-index (1, 3, and 5 years: 0.842, 0.801, and 0.799, respectively). The calibration plot of the nomogram for 1, 3, and 5 years showed optimal agreement between the prediction by the nomogram and the actual observation outcome (Figures 4D–F). Overall, these results suggest that the nomogram was a useful predictor for the survival of patients with HCC.

### Functional Analyses Based on the Risk Model

Overall, 58 pyroptosis-related DEGs were found in this study between tumor tissues and normal tissues (18 were downregulated and 47 were upregulated). GO function enrichment analysis and KEGG pathway enrichment analysis were carried out on pyroptosis-related DEGs in tumor tissues and normal tissues. The GO analysis results indicated that pyroptosis-related DEGs mainly focused on “pyroptosis,” “positive regulation of cytokine production,” “response to molecule of bacteria origin,” “response to lipopolysaccharide,” and “cellular response to biotic stimulus.” The KEGG analysis results showed that pyroptosis-related DEGs were mainly enriched in “salmonella infection,” “pertussis,” “measles,” “necroptosis,” and “lipid and atherosclerosis” (Supplementary Figure S5).

**FIGURE 5:**
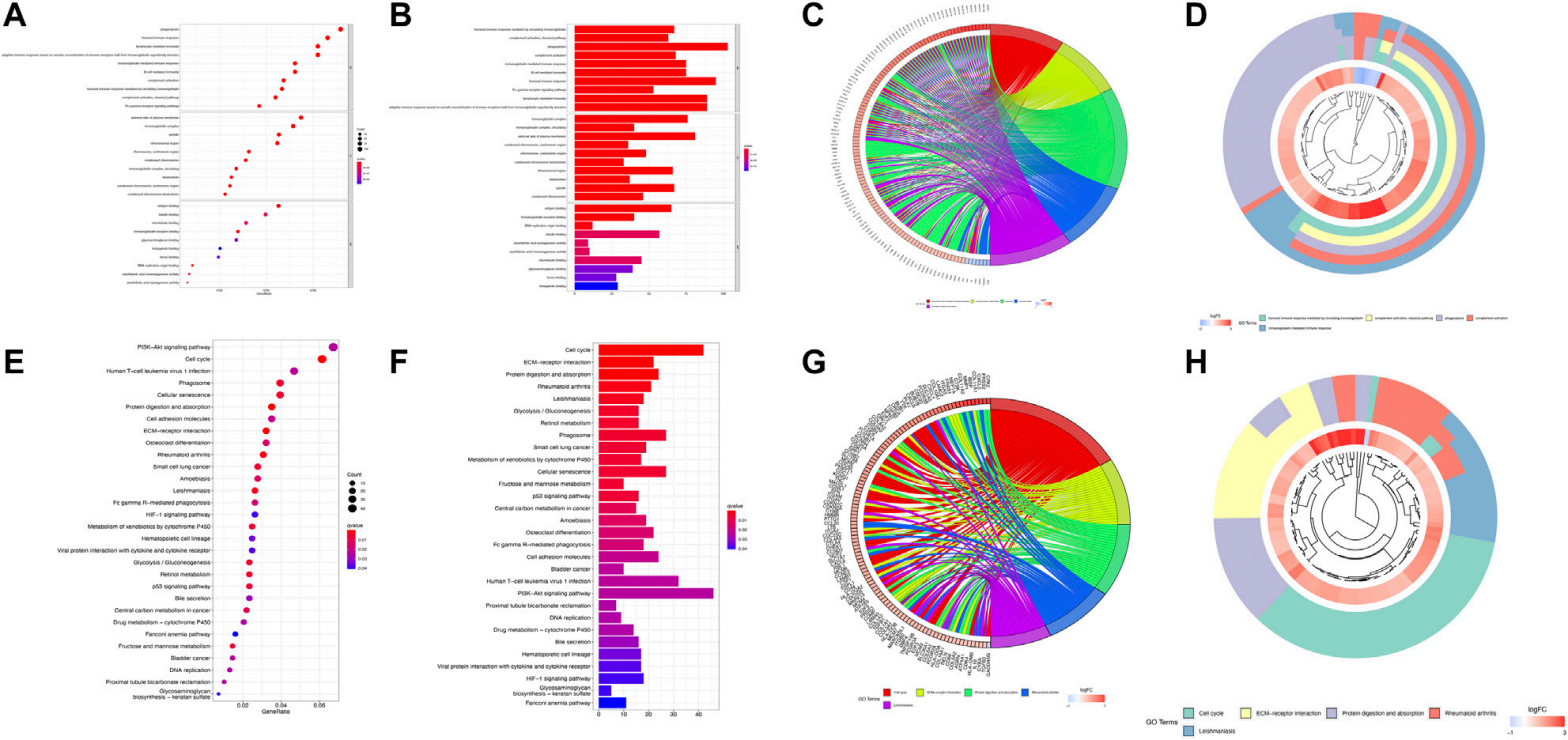
Functional analysis based on the DEGs between the high- and low-risk groups. The bubble plot **(A)**, barplot **(B)**, circular plot **(C)**, and cluster plot **(D)** of the GO pathways enriched for the pyroptosis-related DEGs between the high- and low-risk groups. The bubble plot **(E)**, barplot **(F)**, circular plot **(G)**, and cluster plot **(H)** of KEGG pathways enriched for the pyroptosis-related DEGs between the high- and low-risk groups.

In total, 1,769 DEGs between the low- and high-risk groups were identified. Among them, 1,592 genes were upregulated in the high-risk group, whereas the other 177 genes were downregulated. GO enrichment analysis and KEGG pathway analysis were then performed based on these DEGs. GO enrichment analysis shows that the DEGs are between the high- and low-risk groups mainly focused on “humoral immune response mediated by circulating immunoglobulin,” “complement activation, classical pathway,” “phagocytosis,” “complement activation,” and “immunoglobulin-mediated immune response” for the biological process, whereas “immunoglobulin complex,” “immunoglobulin complex, circulating,” “external side of plasma membrane,” “condensed chromosome, centromeric region,” “chromosome, centromeric region” for cellular component; “antigen binding,” “immunoglobulin receptor binding,” “DNA replication origin binding,” “tubulin binding,” and “arachidonic acid epoxygenase activity” were a focus of molecular function. In addition, the KEGG analysis results show that the DEGs were mainly enriched in pathways associated with “cell cycle,” “ECM–receptor interaction,” “protein digestion and absorption,” “rheumatoid arthritis,” and “leishmaniasis.” We used bar, circular, and cluster plots to show the enrichment pathways involving DEGs and their expression levels, as shown in Figures 5A–H.

**FIGURE 6:**
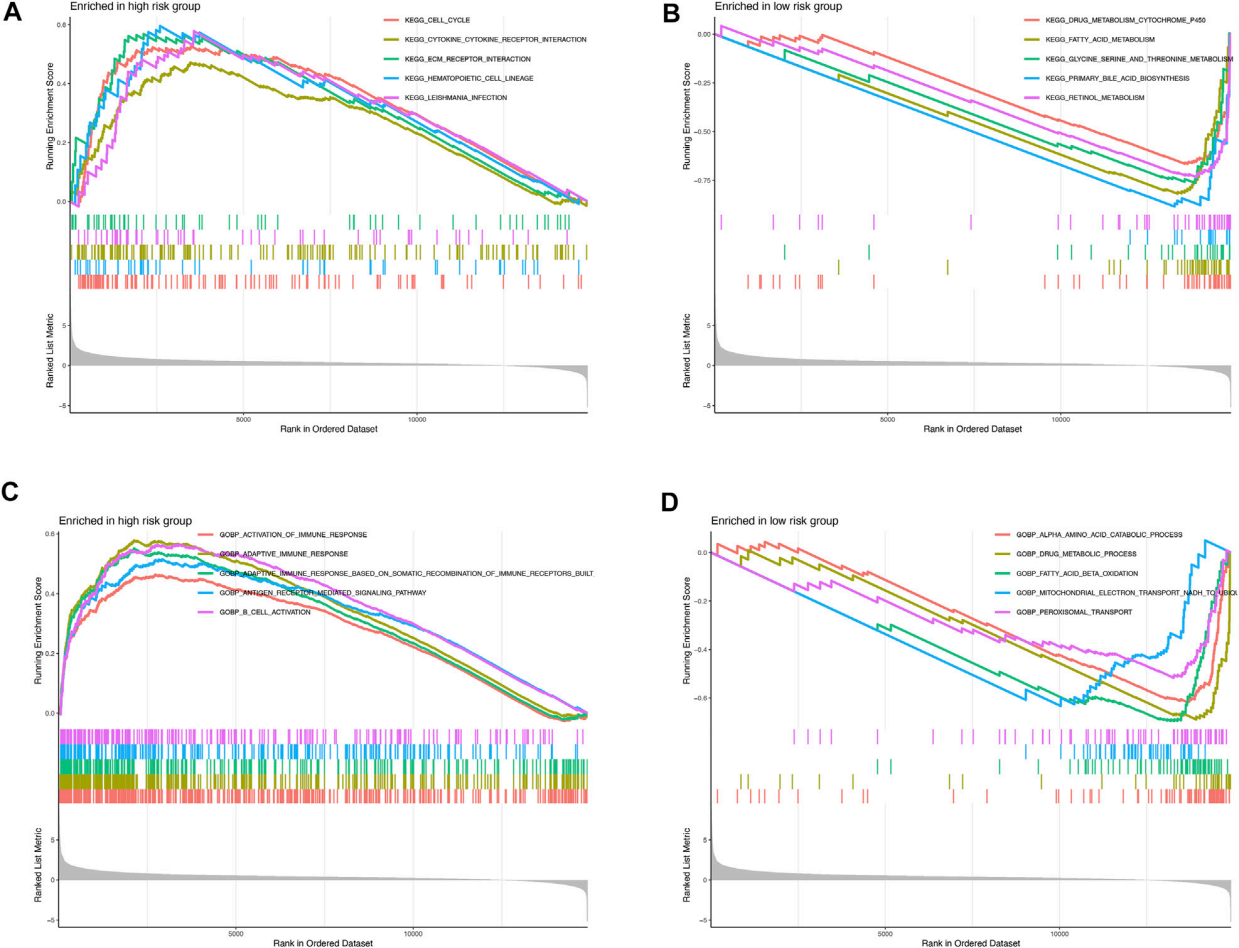
Enrichment plots from gene set enrichment analysis in the high- and low-risk groups **(A,B)** KEGG enrichment analyses; **(C,D)** GO biological processes enrichment analyses.

GSEA was further performed to complement and validate the functional annotation from KEGG and GO functions. KEGG enrichment analyses indicated that the most enriched pathway in the high-risk were cell cycle (NES = 1.763), cytokine–cytokine receptor interaction (NES = 1.605), ECM–receptor interaction (NES = 1.794), hematopoietic cell lineage (NES = 1.847), and *Leishmania* infection (NES = 1.812). In contrast, drug metabolism cytochrome P450 (NES = −3.588), fatty acid metabolism (NES = −3.957), glycine serine and threonine metabolism (NES = −3.413), primary bile acid biosynthesis (NES = −3.311), and retinol metabolism (NES = −3.774) were enriched in the low-risk groups (Figures 6A,B). Furthermore, GO enrichment analyses indicated that the most enriched biological processes in the high-risk were closely associated with immune responses, including the activation of immune response (NES = 1.656), adaptive immune response (NES = 2.059), adaptive immune response based on somatic recombination of immune receptors built from immunoglobulin superfamily domains (NES = 1.933), antigen receptor–mediated signaling pathway (NES = 1.800), B-cell activation (NES = 1.973), and so on. In contrast, α amino acid catabolic process (NES = −3.892), drug metabolic process (NES = −3.163), fatty acid beta oxidation (NES = −3.986), mitochondrial electron transport NADH to ubiquinone (NES = −3.356), and peroxisomal transport (NES = −2.880) were enriched in the low-risk-score group, as shown in Figures 6C,D. Altogether, the above results indicate that the pyroptosis-related lncRNA signature be associated with the immune responses.

### The Landscape of TME Immune Infiltration in HCC

**FIGURE 7:**
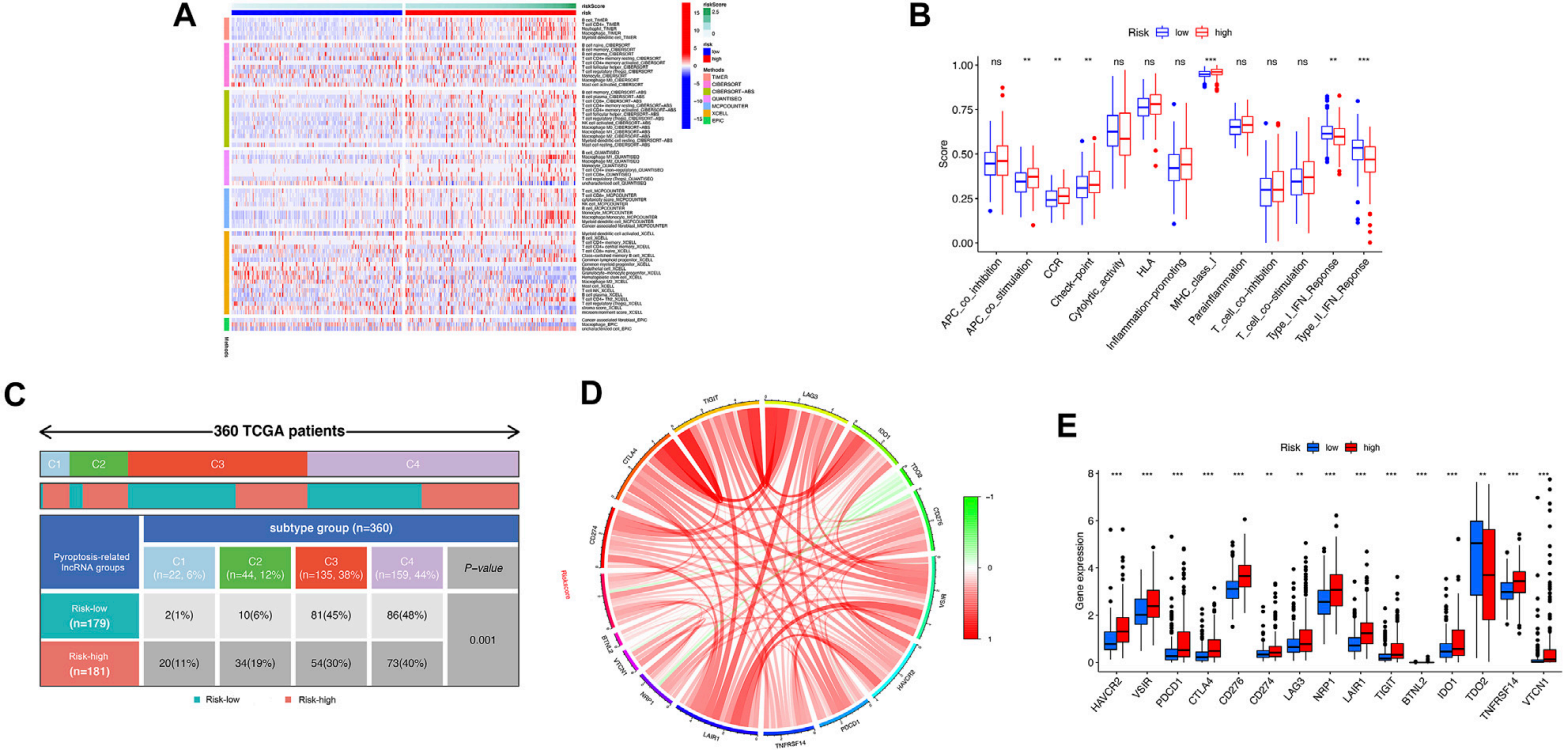
Correlation analysis was performed between the risk model and immune status. **(A)** The association between immune cell infiltration and the risk score using the different algorithms. **(B)** The boxplot illustrating the difference in immune-related functions between the high- and low-risk groups. **(C)** Comparison in the differences in immune subtype between different risk groups. **(D)** Chord diagram illustrating the correlations between the expression of 15 key immune checkpoint genes, as well as between the mRNA expression of 15 key immune checkpoint and the risk score. **(E)** The boxplot displaying the difference in immune checkpoint genes between the high- and low-risk groups. ns, not significant; *p < 0.05; **p < 0.01; ***p < 0.001.

Based on the above functional analyses, we compared the correlation between risk score and immune cells based on different algorithms showing the heatmap of immune responses based on TIMER, CIBERSORT, CIBERSORT-A, QUANTISEQ, MCPCOUNT, XCELL, and EPIC algorithms. Compared with the low-risk groups, the high-risk subgroup generally had high levels of infiltration of immune cells, especially B cells, CD4^+^ T cells, neutrophil cells, and macrophages (M2), as shown in Figure 7A. Subsequently, the ssGSEA algorithm was used to compare the activities of immune-related pathways between the high- and low-risk groups. Immune-related functions, including APC costimulation, CCR, checkpoint, MHC class I, were enriched in the high-risk groups, but types I and II interferon responses were enriched in the low-risk group (Figure 7B). In addition, the proportions of immune subtypes of HCC were compared between different risk groups. The results indicated that there were significant differences in immunophenotyping between high- and low-risk groups (Figure 7C). Higher gene expression levels of NRAV, SNH4, AL031985.3, AL049840.5, AC099850.3, and LINC01224 were found in C1 subtypes, whereas higher expression of AC008549.1 was found in C4 subtypes. The expression of MKLN1-AS did not differ significantly between these subtypes (*p* > 0.05). Patients with HCC in C1 the subtype had the highest risk score, followed by C2 and C4; C3 has the lowest risk score (Supplementary Figure S6). We further studied and analyzed immune checkpoint inhibitor genes between the different risk groups. Immune checkpoint genes (*PD-L1*, *CTLA4*, *TIGIT*, *LAG3*, *IDO1*, *CD276*, *VSIR*, *HAVCR2*, *TNFRSF14*, *LAIR1*, *NRP1*, *BTNL2*, *VTCN1*, and *PDCD1*) were highly expressed in the high-risk group, whereas *TDO2* was highly expressed in the low-risk group, which implies that the high-risk group might have a better response to immunotherapies (Figures 7D,E).

**FIGURE 8:**
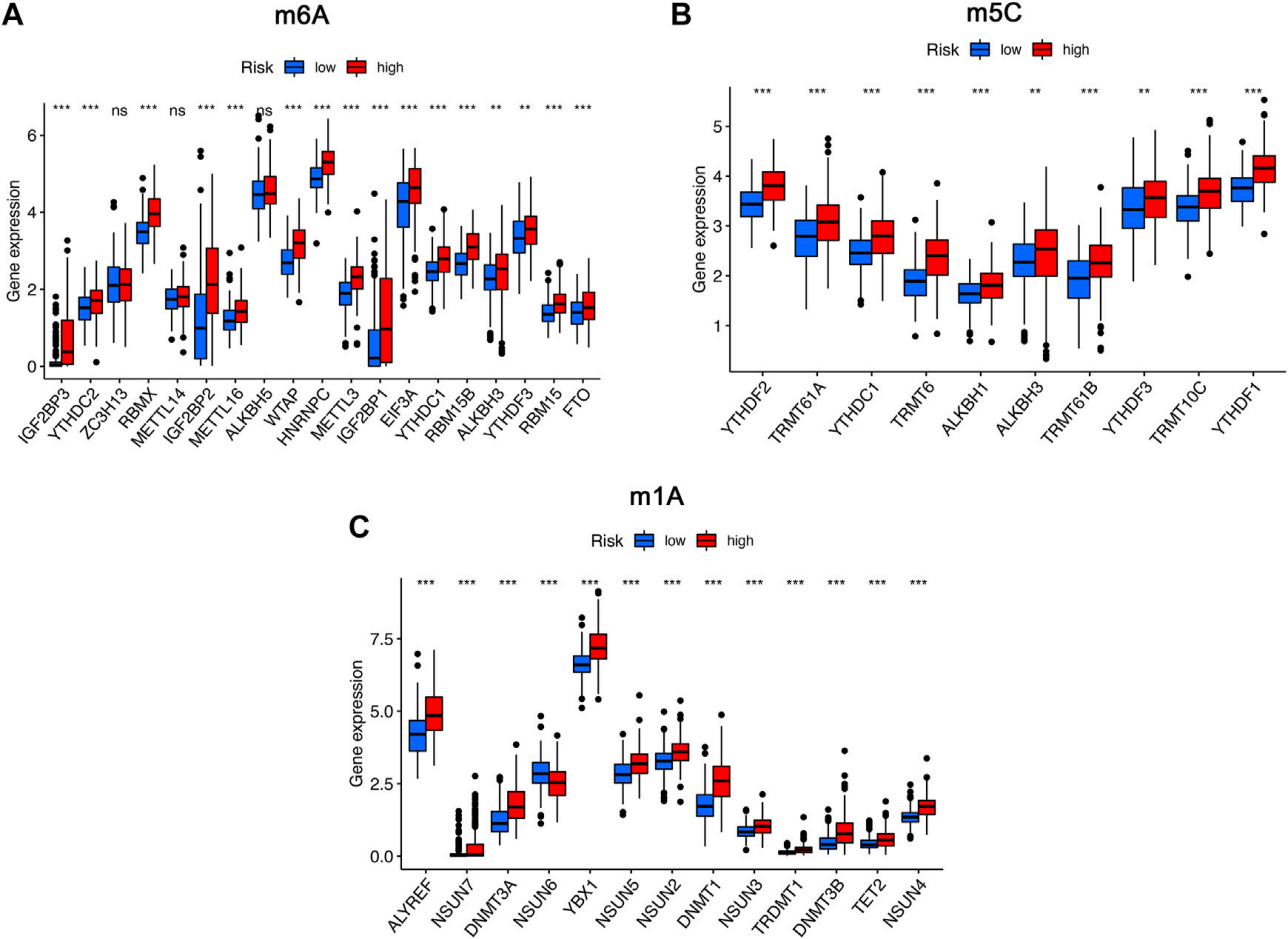
The expression of RNA modification-related genes between the high- and low-risk groups. **(A)** The expression of m6A-related genes between the high- and low-risk groups. **(B)** The expression of m5C-related genes between the high- and low-risk groups. **(C)** The expression of m1A-related genes between the high- and low-risk groups. ns, not significant; *p < 0.05; **p < 0.01; ***p < 0.001.

To further analyze the influence of RNA epigenetic modification on tumorigenesis and tumor progression, the expression of key RNA modification genes was compared across different risk groups. We found that the expression of IGF2BP3, YTHDC2, *RBMX*, *IGF2BP2*, *METTL16*, *WTAP*, *HNRNPC*, *METTL3*, *IGF2BP1*, *EIF3A*, *YTHDC1*, *RBM15B*, *YTHDF3*, *RBM15*, and FTO was significantly higher in the high-risk group than in the low-risk group for m6A modification (Figure 8A). Similarly, the expression of *YTHDC1*, *TRMT61A*, *YTHDC1*, *TRMT6*, *ALKBH1*, *ALKBH3*, *TRMT61B*, *YTHDF3*, *TRMT10C*, and *YTHDF1* in the high risk was higher than low-risk group for m1A modification (Figure 8B). Comparison of m5C-related mRNA expression between the high- and low-risk groups suggested that the expression of ALYREF, NSUN7, *DNMT3A*, *NSUN6*, *YBX1*, *NSUN5*, *NSUN2*, *DNMT1*, *NSUN3*, *TRDMT1*, *DNMT3B*, *TET2*, and *NSUN4* was significantly different, and expression of the above genes was also higher in the high-risk group than in the low-risk group (Figure 8C).

### Correlation Analyses of the Risk Score and Drug Susceptibility

**FIGURE 9:**
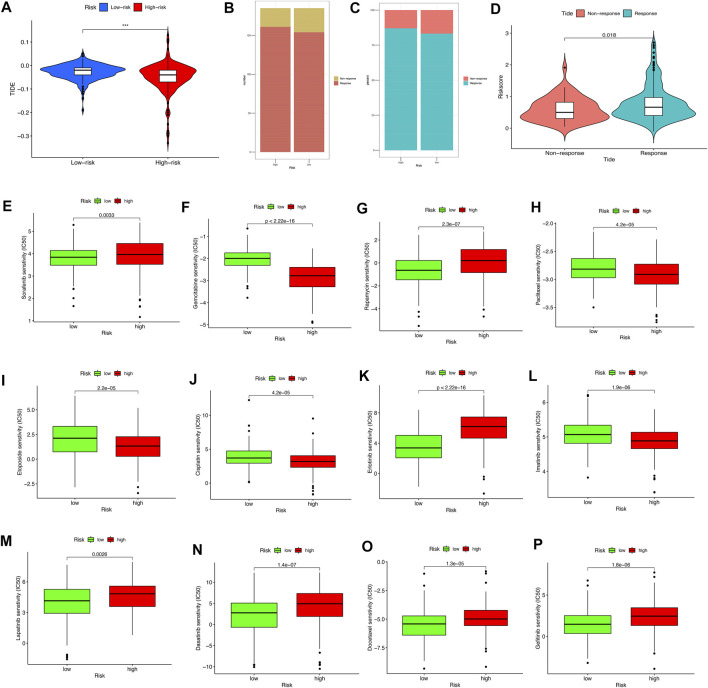
The relationship between the different risk groups and chemotherapeutic efficacy. **(A)** Comparison of the Tumor Immune Dysfunction and Exclusion (TIDE) prediction scores in the low- and high-risk groups. **(B,C)** Distribution and percentage of immunotherapy response among risk groups of HCC patients. **(D)** Comparison of risk scores between the response group and the nonresponse group. **(E–P)** Sensitivity analysis of drugs between different risk groups.

The TIDE algorithm was utilized to explore whether the pyroptosis-related lncRNA risk score model could predict the immunotherapeutic benefit in HCC patients. The results indicated that the TIDE prediction score in the high-risk group was significantly lower than that in the low-risk group (Figure 9A); furthermore, the number of immunotherapy responders in the high-risk group was significantly higher than that in the low-risk group (*p* < 0.05, Figures 9B,C). After grouping the degree of response to immunotherapy, we found that the response group had a higher risk score than the nonresponse group (Figure 9D). The above results highlighted that the high-risk group might have a better treatment prospects for immunotherapy.

Notably, the sensitivity of HCC to various chemotherapeutic drugs is relatively poor, which limits its wide application. To select individualized chemotherapy for patients, we evaluated the IC_50_’s of various chemotherapy drugs between the high- and low-risk groups. The results of drug susceptibility indicated that the high-risk group had lower IC_50_’s of gemcitabine, paclitaxel, etoposide, cisplatin, and imatinib than those in the low-risk group, which suggests that patients at high risk may benefit more from the above chemotherapy, whereas the IC_50_ of sorafenib, rapamycin, erlotinib, lapatinib, dasatinib, and gefitinib in the low-risk group is lower than that of the high-risk group, which suggests that low-risk group patients might obtain survival benefits from the choice of chemotherapy drugs mentioned previously (Figures 9E–9P).

## Discussion

HCC is the most common primary liver cancer worldwide. Because of its characteristics of rapid tumor progression, recurrence, metastasis, and resistance to treatment, its prognosis is usually very poor. HCC, as a leading cause of cancer-related mortality worldwide, has a significant influence on the quality of life of HCC and also incurs huge economic costs (Jemal et al., 2011; Sempokuya et al., 2021). Although many recent efforts have been made to treat HCC, the long-term prognosis of HCC patients remains very poor. Meanwhile, the pathogenesis of HCC is still unclear, there is a lack of novel molecular biomarkers to predict the prognosis of HCC patients and distinguish patients with potential sensitivity to various treatments. Given its high mortality rate, it is urgent to find new therapeutic targets that are closely related to the prognosis of HCC patients.

Pyroptosis, as a newly discovered type of programmed cell death, has recently attracted increasing attention in the field of cancer biological research (Du et al., 2021; Loveless et al., 2021). Increasing evidence has shown the important dual role and mechanisms of pyroptosis involved in the development and treatment of various cancers (Cai et al., 2021; Guo et al., 2021c; Shao et al., 2021; Ye et al., 2021; Zhang et al., 2021). On the one hand, pyroptosis can cause excessive inflammation in the body, release many inflammatory cytokines, and change the immune microenvironment, thereby promoting the generation and development of tumors (Karki and Kanneganti, 2019). On the other hand, pyroptosis can help cells maintain homeostasis, improve immune activity, promote tumor cell pyroptosis, and protect the host (Ruan et al., 2020). Therefore, pyroptosis could become a promising new therapeutic target in the process of cancer treatment. However, the specific function of pyroptosis has been less well studied in HCC. Numerous studies have suggested that lncRNAs play a key role in the occurrence, development, and metastasis of liver cancer, and they have shown strong survival prediction potential as novel biomarkers (Hong et al., 2020; Li et al., 2020; Wan et al., 2021; Zhou et al., 2021). For example, Cao reported that lncRNA TMEM220-AS1 could suppress HCC cell proliferation and metastasis by regulating the miR-484/MAGI1 axis (Cao et al., 2021). However, the prognostic value and mechanism of pyroptosis-related lncRNAs in HCC have not yet been elucidated and remain to be studied. Furthermore, the potential role of pyroptosis-related lncRNA signatures as an effective therapeutic strategy in HCC is unknown.

Therefore, our study systematically explored the expression of pyroptosis-related genes and their related lncRNAs that affect the prognosis of HCC patients. Moreover, we developed a pyroptosis-related lncRNA signature to provide promising prognostic value biomarkers in HCC. Among the identified pyroptosis-related lncRNAs, ZFPM2-AS1, KDM4A-AS1, LUCAT1, NRAV, AL031985.3, AL049840.5, MKLN1-AS, AC099850.3, and LINC01224 are potentially risk lncRNAs, but CRYZL2P-SEC16B, and AC008549.1 are potentially protective lncRNAs. In this study, many lncRNAs in the risk model, such as ZFPM2-AS1, NRAV, LUCAT1, MKLN1-AS, and LINC01224, were found to exert vital roles in regulating and participating in the progression of different cancers (He et al., 2020a; Gao et al., 2020; Liu et al., 2020; Sun et al., 2021). For example, studies have demonstrated that dysregulation of lncRNA ZFPM2-AS1 is implicated in the initiation and development of a variety of malignant tumors; lncRNA ZFPM2-AS1 is located at the 8q23 region, an aggregate of cancer susceptibility loci, (Okamoto et al., 2003), and can act as an oncogene to promote HCC cell proliferation, invasion, and metastasis. As discovered by Xiao et al., lncRNA ZFPM2-AS1 can promote colorectal cancer progression by sponging miR-137 to regulate TRIM24 (Xiao et al., 2021). Similarly, LUCAT1 also plays a key role in the progression and metastasis of HCC. Lou found that lncRNA LUCAT1 could promote tumorigenesis by inhibiting ANXA2 phosphorylation in HCC (Lou et al., 2019). In addition, NRAV, MKLN1-AS, AC099850.3, LINC01224, and AL031985.3 were found to be also closely related to immunity, autophagy, and glycolysis in HCC, suggesting a possible association between pyroptosis and the aforementioned immune and metabolic regulation in cancer and may represent an oncogene that predicts HCC prognosis. To date, lncRNAs (CRYZL2P-SEC16B and AL049840.5) have not been reported in the literature, and there is a lack of research on the contribution to the prognostic value of cancer and pyroptosis. Therefore, more in-depth studies are needed to explore the role of these lncRNAs in HCC and their impact on the pyroptosis of liver cancer cells.

In our study, Kaplan–Meier survival analysis showed that high-risk group was associated with worse OS. We further analyzed and explored the association between the risk score and clinical characteristics. As expected, we found that the pyroptosis-related risk score was significantly positively correlated with tumor stage, pathological grade, and tumor-node-metastasis (TNM) stage. A higher risk score was associated with a higher tumor pathological grade, more advanced TNM staging, and worse patient prognosis. Through ROC curve analysis, we found that our pyroptosis-related lncRNA score model had good sensitivity and specificity, and its predictive power was significantly higher than that of other clinicopathological indicators. We established a nomogram based on the risk score combined with other clinicopathological factors to predict the survival rate of HCC patients. The C-index of the OS prediction was 0.756 (95% CI, 0.714–0.798). The calibration plot for the probability of OS at 1, 3, or 5 years showed an optimal agreement between the nomogram prediction and the actual observation.

To further explore the potential functional changes between different risk groups, functional enrichment analysis was performed. Compared with the low-risk group, highly expressed genes in the high-risk group were focused on cell cycle, cytokine–cytokine receptor interaction, and some immune-related response signal pathways. In addition, we found significant changes in the abundance of immune cells between the high- and low-risk groups. HCC patients in the high-risk group had high levels of infiltration of immune cells, including B cells, CD4^+^ T cells, neutrophils, macrophages, and myeloid dendritic cells. This suggests that higher levels of immune cell infiltration in the high-risk group may lead to worse prognosis; this may be related to the activation of inflammasome-mediated cell pyroptosis and the release of pyroptosis-induced cytokines that change the immune microenvironment and promote tumor development by evading immune surveillance. In addition, this severe chronic inflammation caused by pyroptosis inhibits the antitumor immune response mediated by cells such as M1 macrophages, natural killer cells, and CD8 T cells. Tumor cells themselves also directly suppress tumor immunity by recruiting specific immune cell subsets, such as activating myeloid-derived suppressor cells, M2 macrophages, and regulatory T cells. The above results indicate that pyroptosis might regulate the composition of immune cells and affect the tumor immune environment.

Immunotherapy related to immune checkpoints is a promising method to treat a variety of malignant tumors, and pyroptosis can act synergistically with immune checkpoint suppression to trigger a protective immune response. The latest research has found that pyroptosis in mouse colon cancer and melanoma cells does not affect tumor growth; it can significantly enhance the suppression of tumor growth achieved by blocking immune checkpoints with anti–PD-1 antibodies (Zhou et al., 2020a). Similarly, in our study, it is worth noting that there was a positive correlation between the pyroptosis risk score and almost all immune checkpoint genes. The above results indicate that patients in the high-risk group may benefit more from immunotherapy such as anti–PD-L1 and CTLA-4 treatments. The TIDE algorithm also proves that high-risk patients seem to respond more positively to immunotherapy. The above results show the potential of the pyroptosis-related lncRNA model in predicting the response of HCC patients to immunotherapy. In addition, drug sensitivity analysis also shows the potential benefits of chemotherapy for HCC patients in different risk groups in our study. Previous studies have found that chemotherapeutic drugs can induce or regulate the pathway of cell pyroptosis and inhibit tumor growth. For example, studies have found that the chemotherapy drug cisplatin induces pyroptosis in A549 lung cancer cells via caspase 3/GSDME activation (Zhang et al., 2019). Peng et al. also found that GSDME can trigger antitumor immune cell infiltration by mediating pyroptosis and enhance the sensitivity of cisplatin to non–small cell lung cancer (Peng et al., 2020). Our risk score can be an effective biomarker for predicting the effectiveness of chemotherapy in HCC patients, and it can provide new insights for the choice of chemotherapy drugs for HCC patients. It can also provide help to study the correlation between chemotherapy drugs and pyroptosis in HCC. Previous studies have confirmed that the RNA modification can change the structure of lncRNAs and affect their interaction with proteins by interfering with target gene transcription or changing their subcellular distribution (He et al., 2020b). Furthermore, RNA modification can also promote the malignant biological behavior of tumors through signaling pathways and even affect the drug resistance of tumors (Zhou et al., 2020b). Therefore, we compared and analyzed the differences in RNA modification genes between the high- and low-risk groups. Our research will provide new insight into the upstream RNA modification functions and specific regulatory mechanisms of pyroptosis-related lncRNAs.

Although the risk model we established is robust and shows good prospects in predicting the prognosis of HCC patients, this study still has several limitations. First, because we did not find the lncRNA expression profile or gene dataset involved in establishing the pyroptosis-related lncRNA risk score in the Gene Expression Omnibus database and the International Cancer Genome Consortium data portal, only a single TCGA dataset was used. In addition, the TCGA dataset lacks detailed clinicopathological indicators and treatment information, and we therefore could not explore the impact of the above factors on the prognosis of HCC patients. Second, prospective studies are needed to verify the prognostic value of pyroptosis-related lncRNA risk scores. In addition, functional experiments should be performed to further clarify the molecular mechanism of pyroptosis-related lncRNA effects in HCC.

In conclusion, we constructed a pyroptosis-related risk model to predict the OS of HCC patients, and the model showed good predictive ability. In addition, we found that the pyroptosis-related risk score is related to the immune response of HCC patients, which can provide help in the choice of immunotherapy and chemotherapy drugs for HCC patients.

## Data Availability

Publicly available datasets were analyzed in this study. This data can be found here: TCGA (https://portal.gdc.cancer.gov/repository).

## Abbreviations

HCC, hepatocellular carcinoma; lncRNA, Long non-coding RNA; TCGA, The Cancer Genome Atlas; ROC, receiver operating characteristic; TACE, transcatheter arterial chemoembolization; GO, Gene Ontology; KEGG, Kyoto Encyclopedia of Genes and Genomes; FC, fold change; OS, overall survival; GSEA, Gene Set Enrichment Analysis; DEGs, differentially expressed genes; TIDE, Tumor Immune Dysfunction and Exclusion.
