# Supplementary figures and images for: The Pyroptosis-Related Long Noncoding RNA Signature Predicts Prognosis and Indicates Immunotherapeutic Efficiency in Hepatocellular Carcinoma
Source: Front Cell Dev Biol. 2022 May 26;10:779269. doi: 10.3389/fcell.2022.779269 (PMC9195296; doi:10.3389/fcell.2022.779269)

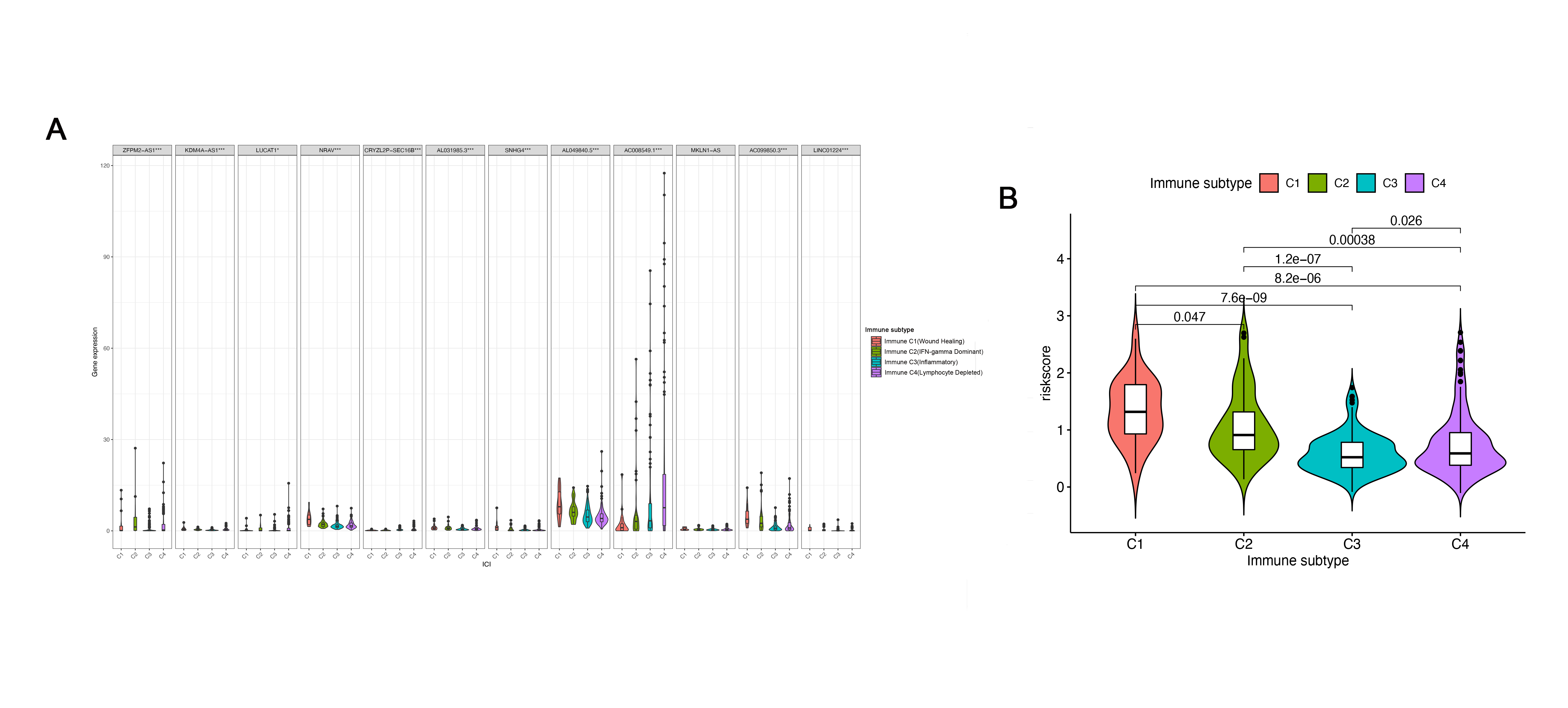

Supplement: Supplementary file 1 [file Image6.TIF]

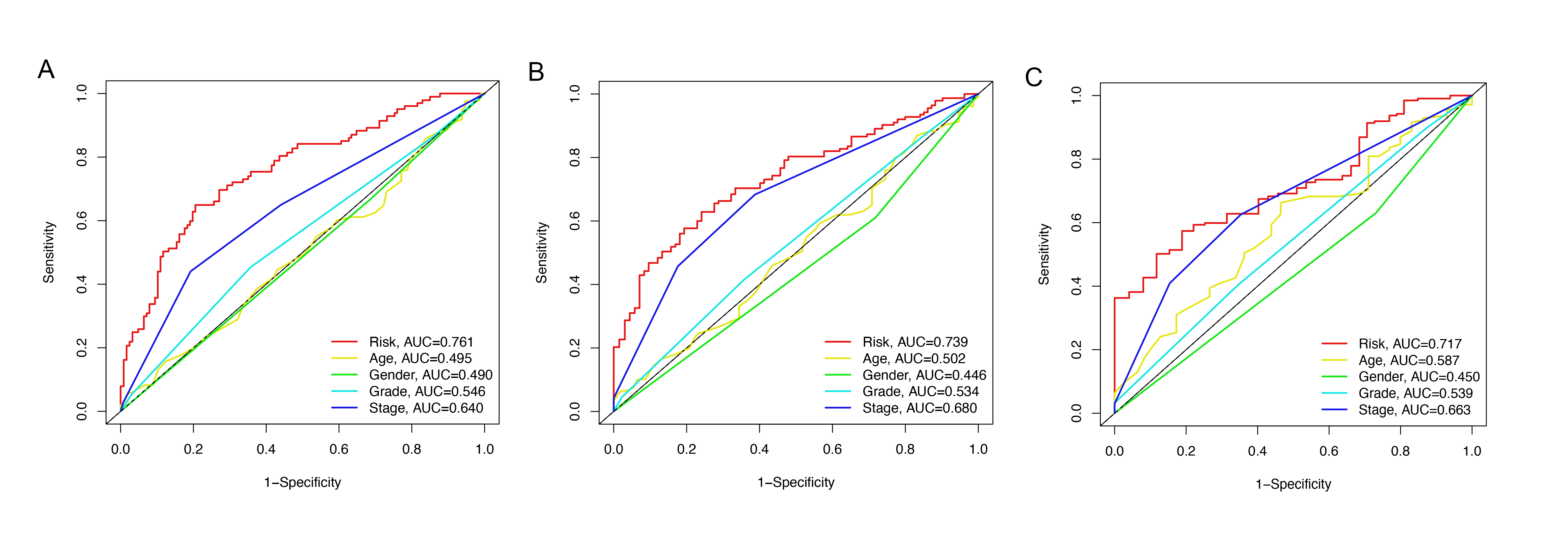

Supplement: Supplementary file 3 [file Image3.TIF]

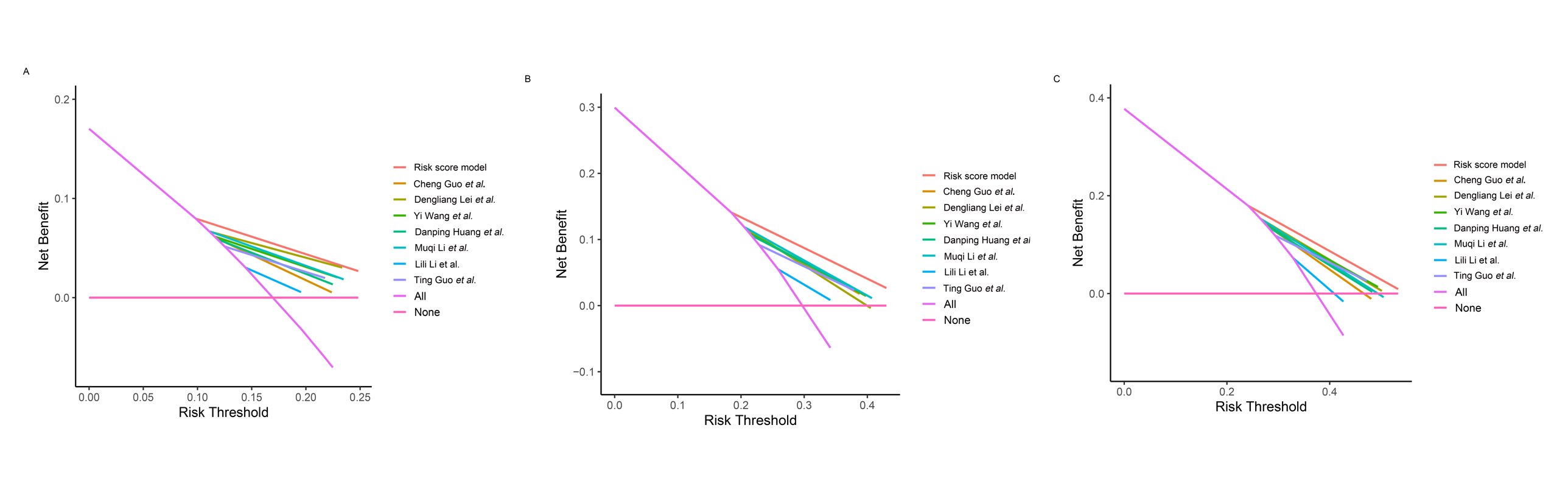

Supplement: Supplementary file 4 [file Image4.TIF]

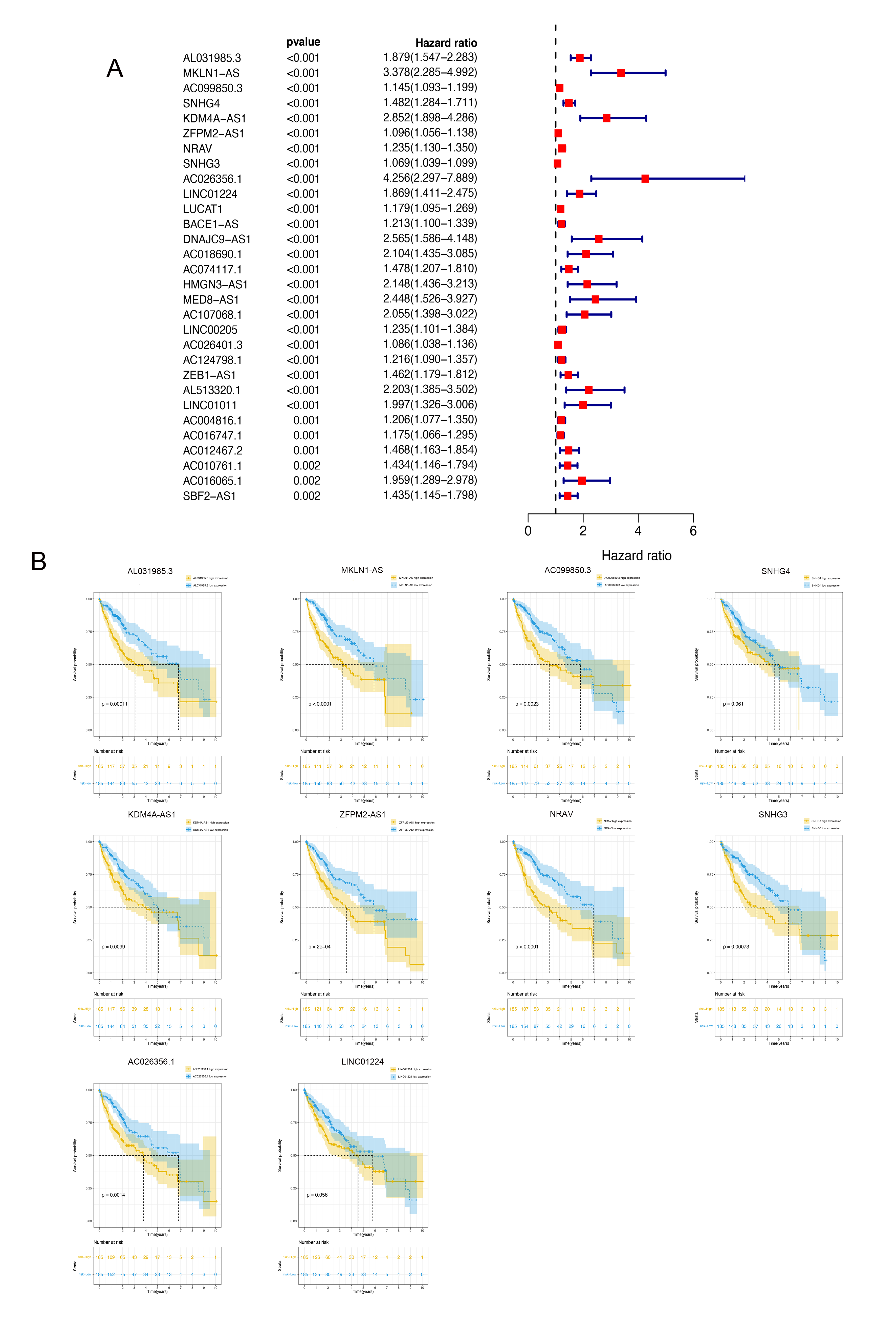

Supplement: Supplementary file 5 [file Image2.TIF]

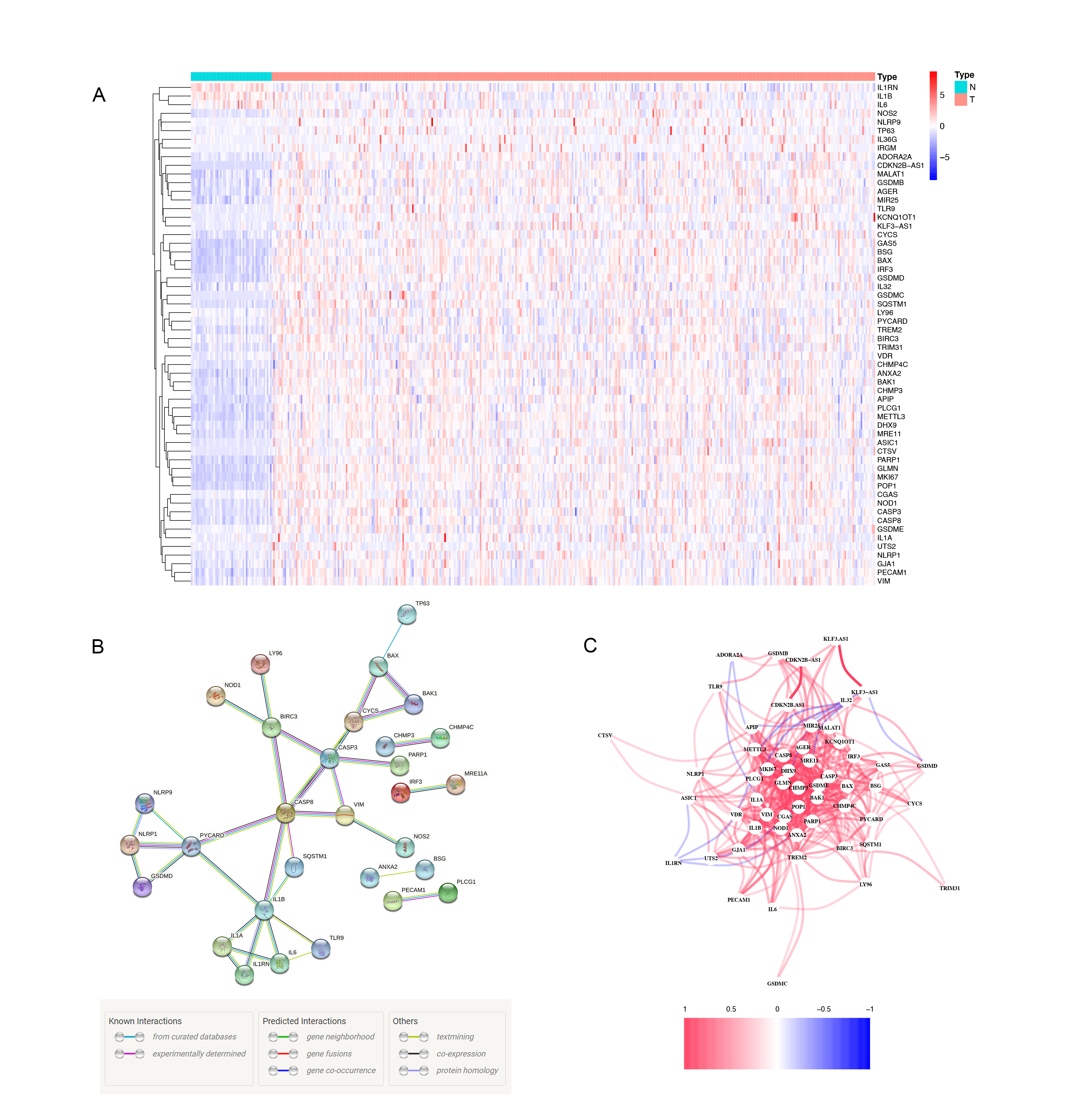

Supplement: Supplementary file 6 [file Image1.TIF]

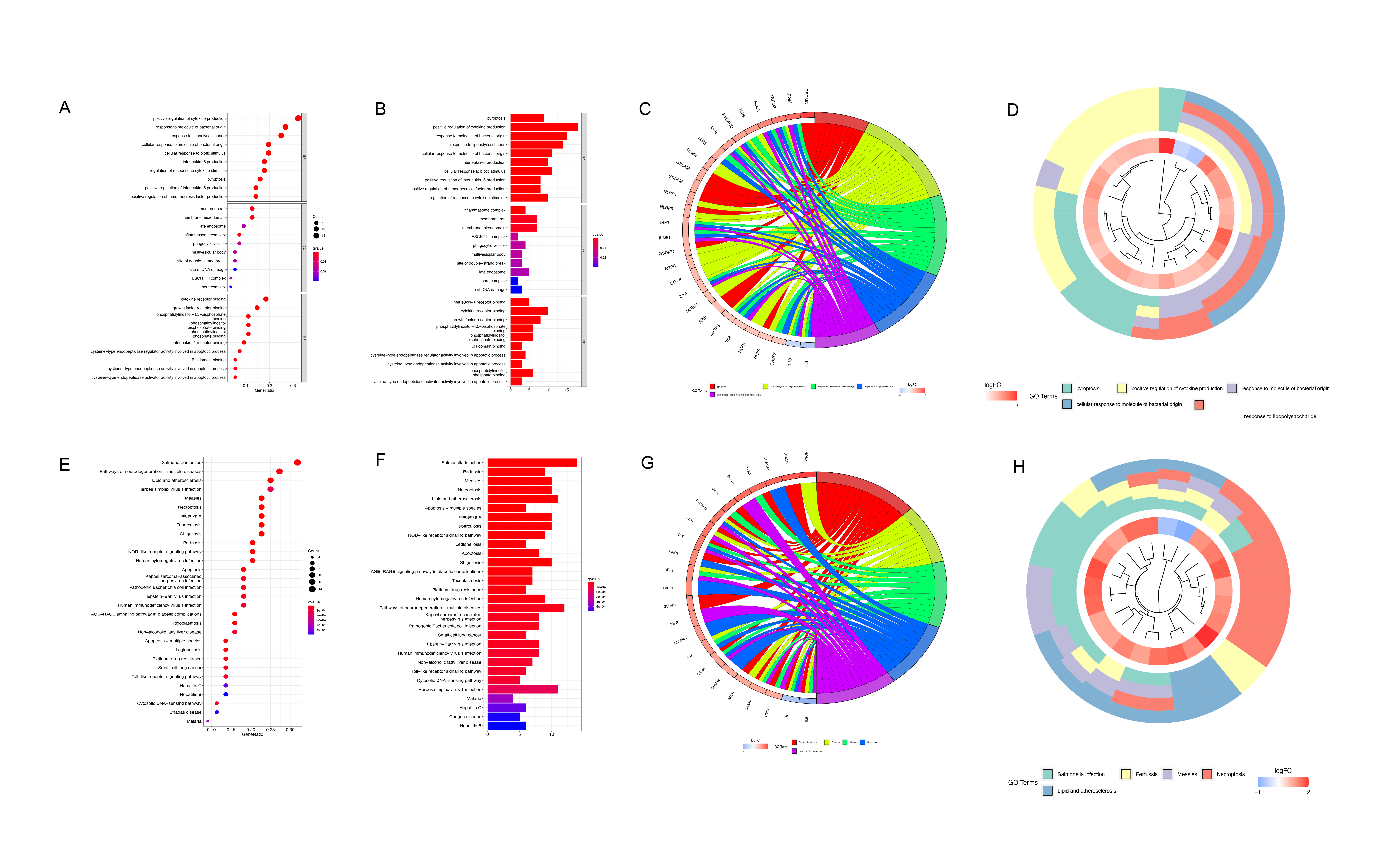

Supplement: Supplementary file 9 [file Image5.TIF]
