# Supplementary material for: The Pyroptosis-Related Long Noncoding RNA Signature Predicts Prognosis and Indicates Immunotherapeutic Efficiency in Hepatocellular Carcinoma
Source: Front Cell Dev Biol. 2022 May 26;10:779269. doi: 10.3389/fcell.2022.779269 (PMC9195296; doi:10.3389/fcell.2022.779269)
Supplement: Supplementary file 7 [file Table2.DOCX]

**Supplementary Table S2 Coefficients for each pyroptosis-related lncRNAs in the LASSO regression models**

| **Affect the prognosis of HCC patients** | | |
| --- | --- | --- |
| **Pyroptosis-related lncRNAs** | | **coefficient** |
| ZFPM2-AS1 | 0.0226 |  |
| KDM4A-AS1 | 0.3518 |  |
| LUCAT1 | 0.0472 |  |
| NRAV | 0.0070 |  |
| CRYZL2P-SEC16B | -0.1110 |  |
| AL031985.3 | 0.1310 |  |
| SNHG4 | 0.0065 |  |
| AL049840.5 | 0.0229 |  |
| AC008549.1 | -0.0063 |  |
| MKLN1-AS | 0.6564 |  |
| AC099850.3 | 0.0330 |  |
| LINC01224 | 0.1888 |  |
