# Supplementary material for: The Pyroptosis-Related Long Noncoding RNA Signature Predicts Prognosis and Indicates Immunotherapeutic Efficiency in Hepatocellular Carcinoma
Source: Front Cell Dev Biol. 2022 May 26;10:779269. doi: 10.3389/fcell.2022.779269 (PMC9195296; doi:10.3389/fcell.2022.779269)
Supplement: Supplementary file 8 [file Table3.DOCX]

**Supplementary Table S3 The improvement of pyroptosis-related lncRNA signature predicting overall survival for HCC according to integrated discrimination improvement (IDI)**

| **Models** | **IDI (95%CI)** | ***P value*** |
| --- | --- | --- |
| Cheng Guo *et al* | 0.066(-0.006-0.144) | 0.066 |
| Dengliang Lei *et al* | 0.040(-0.025-0.109) | 0.252 |
| Yi Wang *et al* | 0.051(-0.016-0.118) | 0.146 |
| Danping Huang *et al* | 0.056(-0.006-0.127) | 0.078 |
| Muqi Li *et al* | 0.042(-0.013-0.106) | 0.162 |
| Lili Li *et al* | 0.102(0.044-0.173) | <0.001 |
| Ting Guo *et al* | 0.065(0.003-0.134) | 0.046 |
